# Intravenous Thrombolysis Administration Rate in Patients With Ischemic Stroke at a Tertiary Private Hospital in Mexico City

**DOI:** 10.7759/cureus.95158

**Published:** 2025-10-22

**Authors:** Luis D Milpas Muñoz, Alonso Gutiérrez Romero, Raul Medina-Rioja, Raúl Anwar Garcia-Santos, Octavio Gonzalez Chon, Dianela Gasca Saldaña

**Affiliations:** 1 Internal Medicine, Médica Sur, Mexico City, MEX; 2 Neurology, Instituto Nacional de Neurología y Neurocirugía Manuel Velasco Suárez, Mexico City, MEX; 3 Neurology, Instituto Nacional de Neurología y Neurocirugía, Mexico City, MEX; 4 Cardiovascular Care, Medica Sur, Mexico City, MEX

**Keywords:** door-to-needle time, intravenous thrombolysis, ischemic stroke, mexico, reperfusion therapy, stroke care quality, stroke registries

## Abstract

Introduction: Ischemic stroke is a major cause of morbidity and mortality in Mexico; however, national intravenous thrombolysis rates remain low compared to international standards.

Objective: To evaluate the rate of intravenous thrombolysis in ischemic stroke patients at a private tertiary hospital in Mexico City and to identify clinical and organizational factors associated with treatment delivery.

Methods: We conducted an observational, retrospective, single-center study including 182 ischemic stroke patients admitted between 2021 and 2023. Demographic, clinical, and imaging data were extracted from medical records. Stroke severity was assessed using the NIHSS. Reperfusion therapies, treatment times (door-to-computed tomography [CT], door-to-needle, and door-to-groin), and reasons for exclusion from thrombolysis were documented. Bivariate and multivariate analyses were performed to identify predictors of thrombolysis.

Results: The mean age was 69.5 years, and 52% (n=95) were men. Hypertension (49.5%, n=90), diabetes (21.4%, n=39), and dyslipidemia (14.3%, n=26) were the most frequent comorbidities. Overall, 39 patients (21.4%) received intravenous thrombolysis, including 13 who also underwent mechanical thrombectomy. Among patients who arrived within 4.5 hours of symptom onset (n=85), 46% (n=39) received thrombolysis; of these, 33 (39% of early arrivers) were treated strictly within the conventional 4.5-hour window. Stroke severity (NIHSS ≥5), anterior circulation involvement, early arrival (<1 hour), concomitant thrombectomy, and shorter door-to-groin times were significantly associated with higher thrombolysis rates. Age, sex, and comorbidities showed no significant association. Only 18% (n=33) of patients achieved a door-to-CT time <25 minutes, 21% (n=38) a door-to-needle time <60 minutes, and 11% (n=20) a door-to-groin time <120 minutes. Hemorrhagic transformation occurred in 20% (n=36), and in-hospital mortality was 9% (n=16), aligning with international registry data.

Conclusions: The thrombolysis rate observed at this tertiary private hospital was higher than national averages, likely due to early recognition of symptoms, access to extended window protocols, and availability of specialized stroke care. Nonetheless, treatment delays remain a significant challenge. Strengthening institutional stroke pathways and reducing door-to-treatment times are key priorities. Broader implementation of streamlined protocols and prehospital notification systems could improve stroke care delivery in middle-income countries like Mexico.

## Introduction

Cerebrovascular disease (CVD) is the second leading cause of death and disability-adjusted life years (DALYs) lost worldwide, surpassed only by ischemic heart disease [1]. In Latin America, the burden of stroke is particularly high, with unfavorable trends compared to high-income countries [2]. In Mexico, the prevalence of stroke has been rising due to population aging, changes in lifestyle, and increased cardiovascular risk factors [3]. According to Instituto Nacional de Estadística y Geografía (INEGI) data from 2021, the mortality rate from stroke in Mexico has decreased by only 15%, unlike the nearly 50% reduction seen in developed countries [4]. Moreover, Mexico ranks among the Latin American nations with the highest stroke-related burden, both in mortality and years of productive life lost [5]. Beyond its clinical impact, stroke imposes a heavy economic burden. A systematic review estimated that the annual cost per patient could reach up to USD 38,000, considering hospitalization, rehabilitation, and productivity loss [6]. 

One of the first epidemiological studies in Mexico, the Brain Attack Surveillance in Durango (BASID) study, reported an incidence of 230 cases per 100,000 inhabitants over 35 years of age and a prevalence of 8 per 1,000 [7]. The RENAMEVASC study, a prospective registry of 2,000 patients, identified cardioembolism (24.7%), small-vessel disease (19.4%), atherosclerosis (14.7%), and undetermined causes (36.6%) as the most frequent etiologies. The most common risk factors in the Mexican population are systemic arterial hypertension, smoking, and type 2 diabetes [8]. More than 50% of patients died or were left with disability. A 25-year registry at the National Institute of Neurology and Neurosurgery confirmed similar patterns, with a predominance of atherosclerosis (21.8%) and cardioembolism (21.2%) [9].

Despite growing awareness of stroke management, multiple barriers delay timely treatment. In a retrospective multicenter study of four hospitals in Mexico, only 17.5% of ischemic stroke patients arrived within the 4.5-hour therapeutic window [10]. The PREMIER study similarly found that just 23% of patients arrived within the first 3 hours [10]. In another study, the average time from symptom onset to hospital arrival was 17 hours. Many patients were initially evaluated in one to four different non-specialized facilities, with delays attributed mainly to inter-hospital referrals (42.6%) and poor recognition of stroke symptoms (34.5%) [11]. Notably, not all patients who arrive within the therapeutic window receive intravenous thrombolysis, often due to contraindications not clearly detailed in available studies [12].

In response, international guidelines have established benchmarks to improve the quality of acute stroke care. Since 2000, recommendations for certified stroke centers have included evaluation within 15 minutes of arrival, neuroimaging within 25 minutes, interpretation within 45 minutes, and administration of thrombolysis within 60 minutes [13]. Certification programs led by the Joint Commission and the American Heart Association have improved stroke care quality and reduced mortality in the U.S. [14]. In Latin America, however, implementation remains limited. The World Stroke Organization (WSO) has proposed a three-tier classification of stroke centers: basic (no imaging or medical staff), essential (CT and thrombolysis capability), and advanced (endovascular therapy and multidisciplinary teams) [15]. In Mexico, some hospitals are now participating in international quality registries such as SITS-QR and Res-Q, which allow monitoring and continuous improvement through key performance indicators [16]. 

Alongside intravenous thrombolysis, mechanical thrombectomy has become an equally crucial reperfusion therapy. Although intravenous thrombolysis has long been the cornerstone of acute ischemic stroke treatment, over the past decade, mechanical thrombectomy has emerged as an essential strategy for patients with large vessel occlusion. This intervention has begun to be integrated into the healthcare systems of middle-income countries such as Mexico. However, its implementation faces challenges similar to and in some cases more complex than those of thrombolysis, including limited access to centers with endovascular capabilities, lack of public healthcare coverage, and the high cost of devices. A recent national study identified that only a fraction of eligible Mexican patients receive this therapy, largely due to the absence of adequate infrastructure and standardized protocols. Therefore, when considering reperfusion strategies, it is essential to jointly address the access challenges of both intravenous thrombolysis and mechanical thrombectomy [17].

To address these challenges and contribute to the national stroke database, we conducted a retrospective registry of ischemic stroke patients at Médica Sur, a private tertiary-level hospital in Mexico City. Using both physical and electronic medical records, we assessed the rate of intravenous thrombolysis, identified factors contributing to treatment variability, and evaluated areas of opportunity. Variability may arise from differences in the implementation of stroke codes, the availability of trained personnel, infrastructure, and hospital protocols. This study seeks to provide evidence that supports the optimization of stroke care in private healthcare settings in Mexico.

Variable definitions and data abstraction

Stroke was defined according to the World Health Organization criteria as a clinical syndrome characterized by the rapid onset of symptoms and/or signs usually corresponding to a focal neurological deficit, and sometimes global (applied to patients with loss of consciousness or acute headache), lasting more than 24 hours or leading to death, with no apparent cause other than a vascular origin. Based on neuroimaging studies (CT or magnetic resonance imaging [MRI]), stroke was classified as ischemic [18,19]. 

To document stroke severity, the National Institutes of Health Stroke Scale (NIHSS) was used, ranging from 0 to 42 points [20].

Symptom-onset-to-door time is defined as the interval between symptom onset (or last known well) and arrival at the emergency department [21].

Door-to-CT time is defined as the interval between arrival at the emergency department and non-contrast cranial CT. The target benchmark is <25 minutes [21].

Door-to-needle time is defined as the interval between arrival at the emergency department and the administration of intravenous thrombolysis. The target benchmark is <60 minutes [21].

Door-to-groin time is defined as the interval between arrival at the emergency department and arterial puncture for mechanical thrombectomy. The target benchmark is <60 minutes [21].

A comprehensive stroke center is defined by the target of achieving >50% of patients with a door-to-needle time <60 minutes and >50% of patients with a door-to-puncture time <120 minutes [21].

Thrombolysis protocol includes alteplase 0.9 mg per kilogram (maximum dose 90 mg), with 10% of the dose administered as an intravenous bolus over 1 minute, followed by the remaining 90% infused over 60 minutes [21].

Thrombolysis rate is defined as the number of patients treated with intravenous thrombolysis divided by the total number of patients with ischemic stroke at the hospital, expressed as a percentage [21].

Eligibility within the thrombolysis window is defined as the number of patients who received intravenous thrombolysis divided by the total number of patients who arrived ≤4.5 hours from symptom onset, expressed as a percentage [21].

Comorbidities

In the present study, the following conditions were considered comorbidities: hypertension, diabetes mellitus, dyslipidemia, atrial fibrillation, smoking, prior stroke or transient ischemic attack (TIA), and obesity: 

Hypertension was defined as a documented diagnosis in the medical record by a treating physician, use of antihypertensive medication at admission, or systolic blood pressure ≥140 mmHg and/or diastolic blood pressure ≥90 mmHg recorded on two or more occasions during hospitalization.

Diabetes was defined as a documented diagnosis in the medical record, current use of antidiabetic medications (oral hypoglycemics or insulin), or hemoglobin A1c ≥6.5% if available.

Dyslipidemia was defined based on documented diagnosis, use of lipid-lowering medications, or LDL cholesterol ≥130 mg/dL.

Atrial fibrillation was defined based on a documented prior diagnosis or electrocardiographic evidence during hospitalization.

Smoking status was defined as current smoking if the patient had smoked within the previous 12 months and former smoking if the patient had quit more than 12 months prior to admission. This information was obtained from the clinical history in the medical record.

Prior stroke or transient ischemic attack (TIA) was defined based on physician-documented diagnosis or neuroimaging evidence of previous infarction.

Obesity was defined as a body mass index (BMI) ≥30 kg/m², as recorded in the admission notes or calculated using height and weight data.

Objectives of the study 

Primary Objective

To determine the rate of intravenous thrombolysis in ischemic stroke patients at Médica Sur Hospital (2021-2023). 

*Secondary Objectives* 

To identify socio-demographic and clinical factors associated with the administration of intravenous thrombolysis. Measure adherence to international time-to-treatment benchmarks (door-to-CT, door-to-needle, door-to-groin). To identify reasons for patient exclusion from reperfusion therapy.

## Materials and methods

An observational, descriptive, retrospective, single-center study was conducted (2021-2023). The medical records of patients admitted to the inpatient and intensive care units of Hospital Médica Sur with a diagnosis of ischemic stroke during the period 2021-2023 were included. No sample calculation was performed; data collection was carried out through consecutive sampling. The study protocol was reviewed and approved by the Institutional Ethics Committee of Médica Sur Hospital. All data were anonymized to ensure patient confidentiality.

Inclusion criteria

Medical records of patients older than 18 years with clinical manifestations of acute ischemic stroke confirmed by neuroimaging (CT or MRI).

Exclusion criteria

1) Medical records of patients who did not meet the World Health Organization definition of ischemic stroke. 2) Medical records of patients with ischemic stroke who were admitted but, for personal reasons (financial, transfer to another center), did not receive treatment or hospitalization at Médica Sur. In addition, medical records whose complete clinical records were not available for data access were excluded.

Data quality and handling of missing values

All data were extracted retrospectively from the hospital’s electronic medical record system using a standardized case report form. To ensure data accuracy, two independent reviewers performed data abstraction, with discrepancies resolved through consensus. Missing data were handled using available-case analysis. Specifically, 26% of patients (n=47) lacked documented onset-to-door times, limiting eligibility assessment for thrombolysis in these cases. These patients were excluded from analyses that required precise time-based eligibility criteria. No data imputation was performed.

Statistical analysis

Bivariate analyses were conducted to explore associations between variables. The chi-square test or Fisher’s exact test was used for categorical variables, as appropriate. For continuous variables, Student’s t-test was applied when normality was assumed, and the Mann-Whitney U test was used otherwise. A multivariable logistic regression model was constructed to identify independent predictors of intravenous thrombolysis and hemorrhagic transformation. Variables with a p-value < 0.1 in bivariate analyses, as well as those deemed clinically relevant based on prior evidence, were included in the multivariable model.

The NIH Stroke Scale (NIHSS) was entered into the model as a continuous variable, given its ordinal nature and clinical interpretation. Model diagnostics were conducted to ensure robustness. Multicollinearity was assessed using variance inflation factors (VIFs); variables with VIF <10 were considered acceptable. The Hosmer-Lemeshow goodness-of-fit test was used to evaluate model calibration, with a p-value >0.05 indicating adequate fit. Results are presented as adjusted odds ratios (aORs) with 95% confidence intervals (95% CIs). All statistical tests were two-tailed, and a p-value <0.05 was considered statistically significant. Data were analyzed using IBM Corp. Released 2025. IBM SPSS Statistics for Windows, Version 31. Armonk, NY: IBM Corp.

## Results

Hospitalization records from January 1, 2021, to December 31, 2023, were reviewed. A total of 194 patients with a diagnosis of ischemic stroke were identified. Of these, 11 patients were excluded for not meeting the World Health Organization definition of ischemic stroke, and one patient was excluded due to the absence of a physical medical record for data collection. Ischemic stroke was confirmed in 182 patient records. 

Sociodemographic and clinical characteristics of the study population

The study included 182 patients with ischemic stroke. The mean age was 69.5 ± 16.7 years, with a median of 73 years (IQR 60.2-81.0). The age distribution was as follows: 33 patients younger than 55 years (18%), 30 patients between 56 and 65 years (16%), 43 patients between 66 and 75 years (24%), 49 patients between 76 and 85 years (27%), and 27 patients older than 85 years (15%). Men represented 51.6% (n=94) and women 48.4% (n=88). Hypertension was the most prevalent comorbidity (49.5%), followed by type 2 diabetes (21.4%) and dyslipidemia (14.3%). Other risk factors included atrial fibrillation (15%), smoking (21%), overweight and obesity (47%), prior ischemic stroke (16%), and transient ischemic attack (24%).

Stroke severity and affected territories

**Figure 1:**
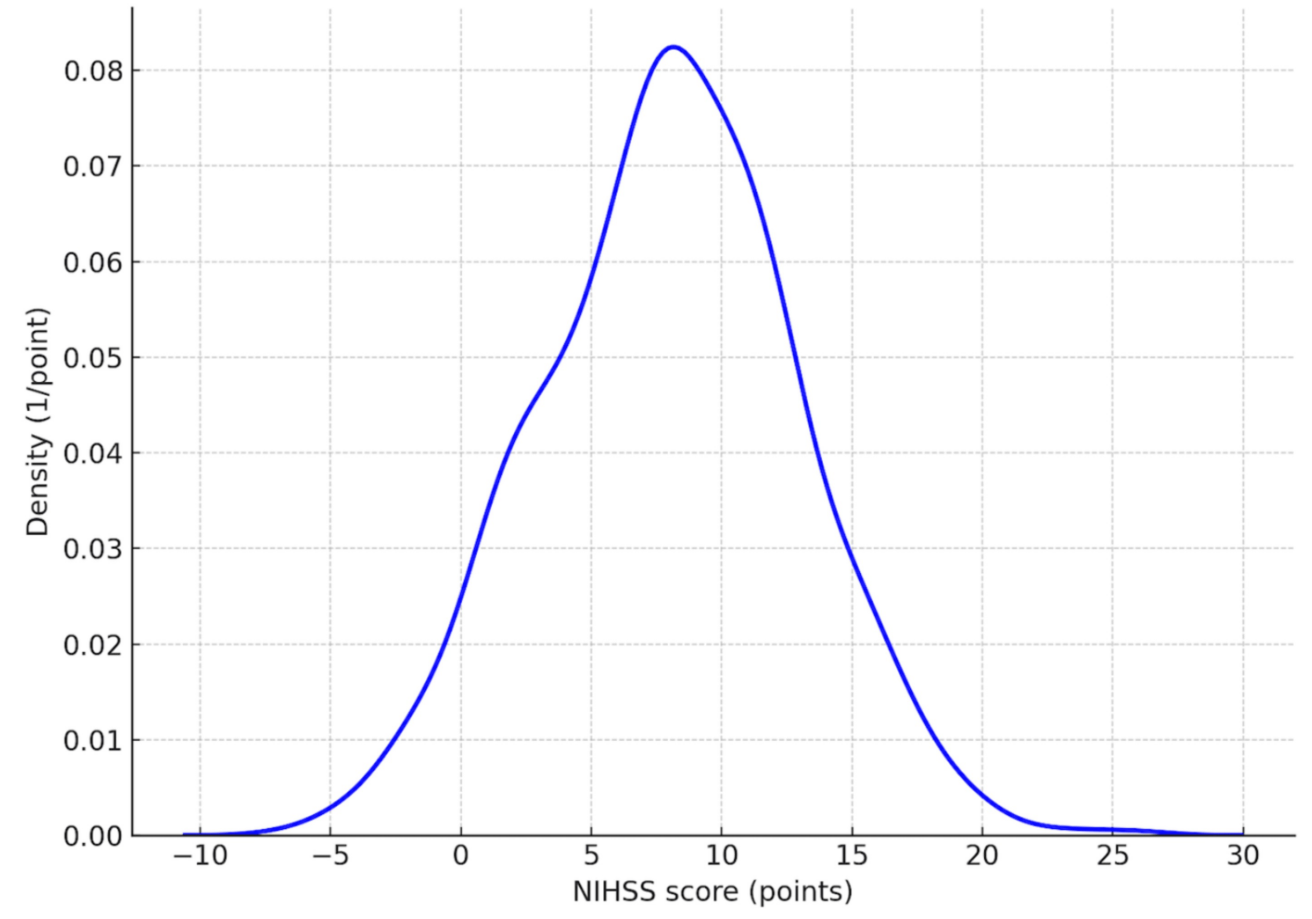
Distribution of NIHSS scores at admission. Density plot showing the distribution of stroke severity according to the NIHSS in the study cohort (n = 182). Figure created by the authors using study data. NIHSS: National Institutes of Health Stroke Scale

The median NIHSS score at admission was 7 points (IQR 2-10). Regarding stroke severity categories, 89 patients (49%) presented with NIHSS 0-4, 67 patients (37%) with NIHSS 5-15, nine patients (5%) with NIHSS 16-20, and 17 patients (9%) with NIHSS >20 (Figure 1).

Concerning vascular distribution, 114 patients (63%) had anterior circulation infarcts, 59 patients (32%) had posterior circulation infarcts, and nine patients (5%) had involvement of both territories. The most frequently affected artery was the middle cerebral artery (MCA) in 101 patients (55.4%), followed by the posterior cerebral artery in 15 patients (8.2%), cerebellar arteries (superior cerebellar artery [SUCA], anterior inferior cerebellar artery [AICA], posterior inferior cerebellar artery [PICA]) in 25 patients (13.7%), vertebral arteries in 10 patients (5.4%), the anterior cerebral artery in three patients (1.6%), the basilar artery in three patients (1.6%), and the artery of Percheron in two patients (1%). 

Onset-to-door time

**Figure 2:**
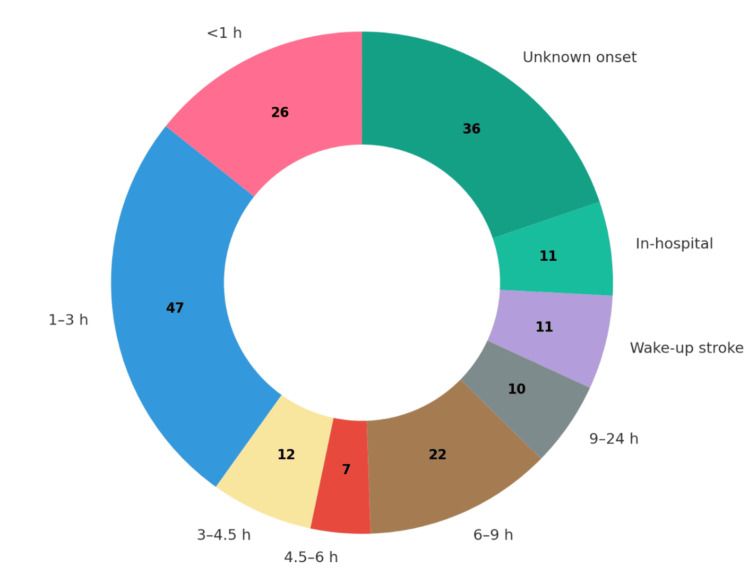
Distribution of onset-to-door time in 182 patients with ischemic stroke. Donut chart illustrates the time interval between symptom onset (or last known well) and arrival at the emergency department. Each colored segment represents a specific onset-to-door time category, with the number of patients labeled at the center of each slice. The largest group arrived between 1 and 3 hours (n = 47), followed by unknown onset (n = 36) and arrival between 6 and 9 hours (n = 22). Other categories included <1 hour (n = 26), 3–4.5 hours (n = 12), 4.5–6 hours (n = 7), 9–24 hours (n = 10), wake-up stroke (n = 11), and in-hospital stroke (n = 11). Figure created by the authors using study data.

Of the 182 patients included, information on symptom onset-to-door time was available for 135 patients (74%). The median time was 294 minutes (IQR 82-403 minutes). Among these, 85 patients (47%) arrived within 270 minutes (4.5 hours) of symptom onset. In addition, 11 patients (6%) presented with in-hospital stroke, 11 patients (12%) with wake-up stroke, and 36 patients (30%) with unknown time of onset (Figure 2). 

Reperfusion therapies 

**Figure 3:**
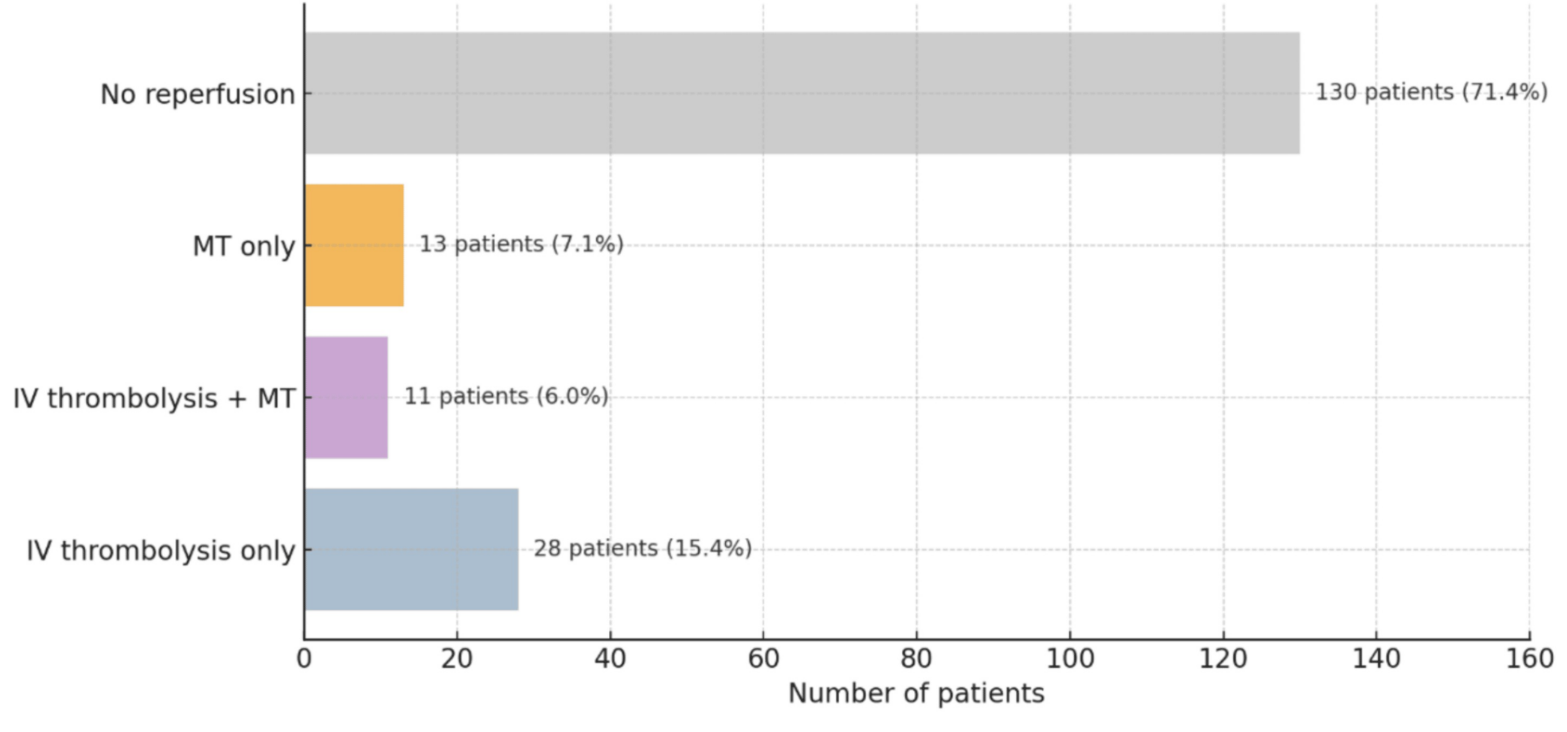
Distribution of reperfusion therapies in patients with acute ischemic strokes (n = 182). Each bar shows the number and percentage of patients who received a specific treatment: IV thrombolysis only includes patients treated exclusively with intravenous thrombolysis. IV thrombolysis + MT refers to those who also underwent mechanical thrombectomy. MT only includes patients who received mechanical thrombectomy without prior thrombolysis. No reperfusion represents patients who did not receive any reperfusion therapy. Figure created by the authors using data from the study.

Of the 182 ischemic stroke patients, 85 (47%) arrived within 4.5 hours of symptom onset. Among these, 39 patients received intravenous thrombolysis, resulting in a thrombolysis rate of 46% among early arrivers. However, only 33 of these 39 patients (85%) were treated strictly within the conventional 4.5-hour window, corresponding to 39% of all patients who arrived within 4.5 hours (Figure 3). Overall, 39 patients (21.4%) received intravenous thrombolysis out of the total cohort (n=182), including 13 who also underwent mechanical thrombectomy. 

Door-to-CT, door-to-needle, and door-to-groin times

**Figure 4:**
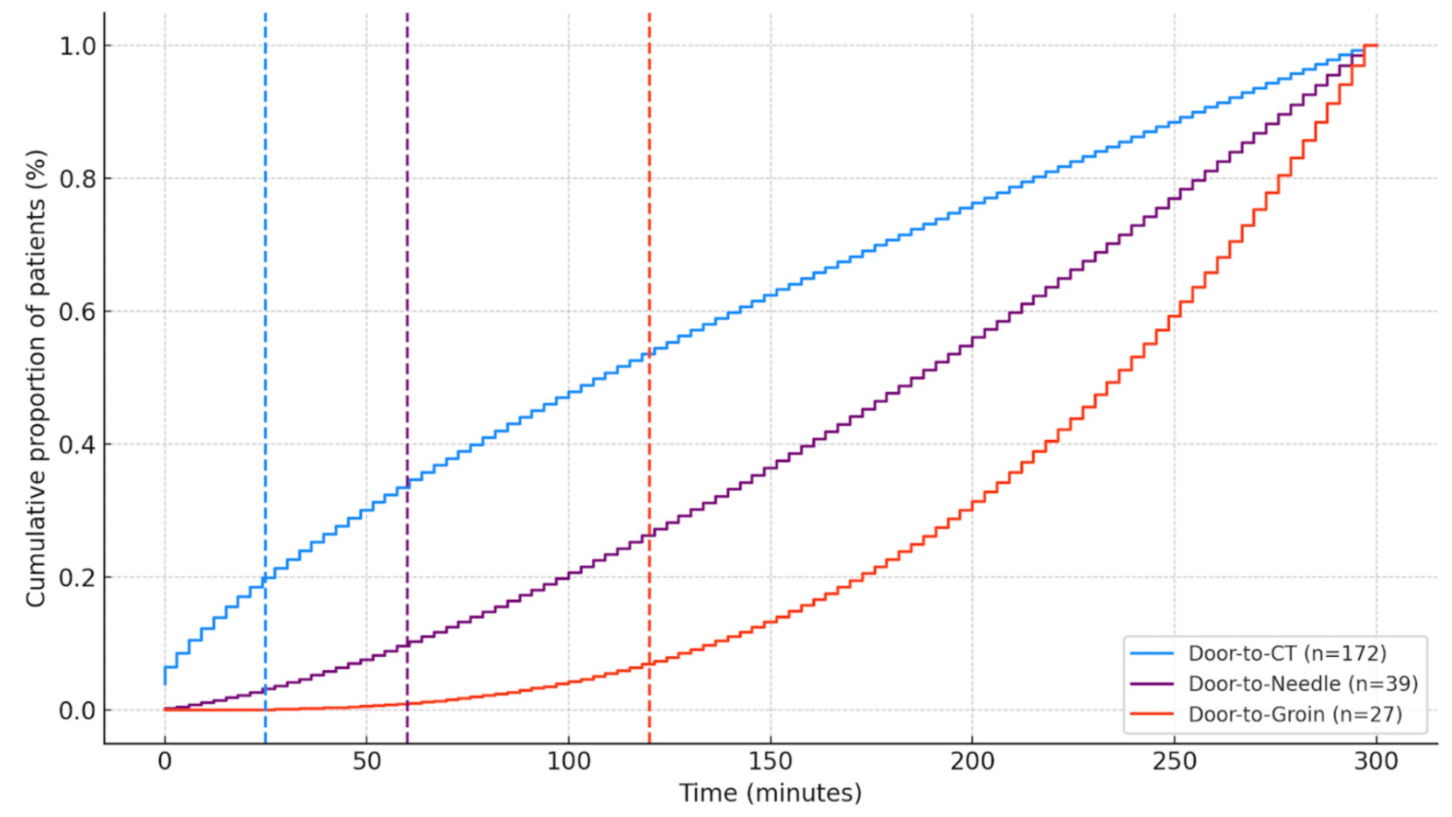
Cumulative distribution of critical times. Cumulative frequency curves of door-to-CT (n=172), door-to-needle (n=39), and door-to-groin (n=27) times are shown. Dashed vertical lines indicate international benchmark targets (<25, <60, and <120 minutes, respectively). The curves illustrate that only a minority of patients achieved recommended time goals, with most undergoing imaging after 60 minutes, thrombolysis after 90 minutes, and mechanical thrombectomy after 180 minutes. Figure created by the authors using study data.

Door-to-CT time was available for 172 patients, with a mean of 121 minutes (IQR 33-104). Among these, 31 patients underwent neuroimaging within <25 minutes (18%), 52 patients between 26 and 60 minutes (29%), and 94 patients after 60 minutes (53%) (Figure 4). Door-to-needle time was documented in 39 patients who received intravenous thrombolysis, with a mean of 112 minutes (IQR 68-150). Five patients were treated within <45 minutes (13%), three patients between 46 and 60 minutes (8%), seven patients between 61 and 90 minutes (18%), and 24 patients >90 minutes (62%). Door-to-groin time was available for 27 patients who underwent mechanical thrombectomy, with a mean of 215 minutes (IQR 150-245). Of these, three patients (11%) underwent groin puncture within <120 minutes, nine patients (33%) between 120 and 180 minutes, and 15 patients (56%) after >180 minutes.

Exclusion reasons for intravenous thrombolysis

**Figure 5:**
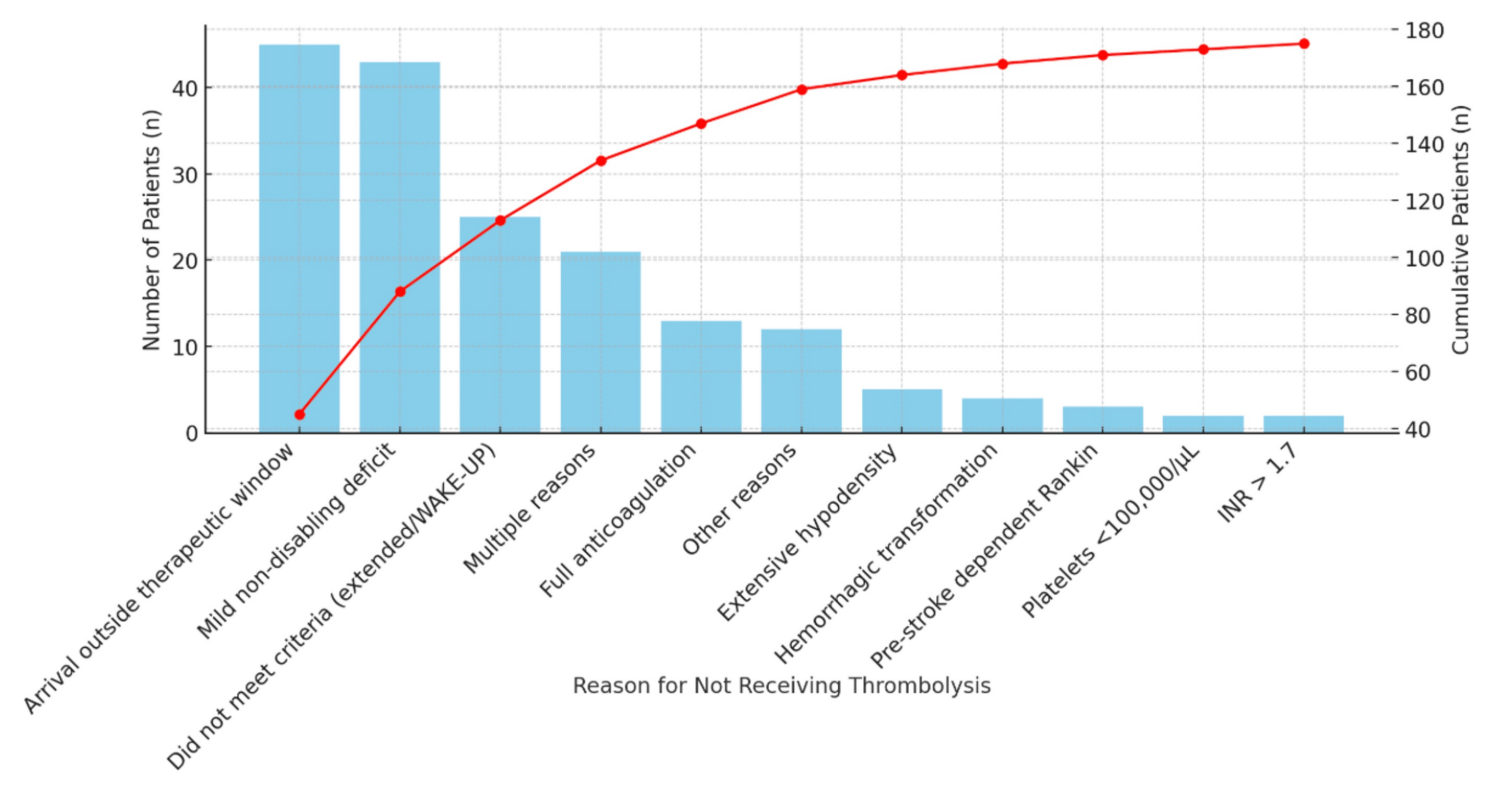
Pareto chart of exclusion reasons for intravenous thrombolysis. The bars represent the absolute number of patients for each exclusion reason, while the red line shows the cumulative percentage. The trend shows a steep initial rise, indicating that the first few exclusion reasons account for the majority of cases. After the first 4-5 categories, the curve flattens, suggesting that the remaining reasons contribute minimally to the overall exclusions. This trend highlights the most impactful exclusion factors, especially: arrival outside the therapeutic window, mild non-disabling deficit, not meeting criteria (e.g., extended/WAKE-UP). Figure created by the authors using study data.

Reasons for exclusion from intravenous thrombolysis were as follows: 45 patients due to arrival outside the therapeutic window (31%), four patients because of extensive hypodensity on neuroimaging (2.7%), one patient due to known platelet count <100,000/µL (0.7%), one patient with INR >1.7 (0.7%), 10 patients under full anticoagulation (6.9%), 25 patients due to mild non-disabling deficits (17%), and 23 patients who did not meet criteria for reperfusion in the extended window or WAKE-UP protocol (16%). In addition, three patients were excluded for hemorrhagic transformation (2%), three for pre-stroke dependent Rankin score (2%), 18 patients presented more than one exclusion criterion (13%), and nine patients had other reasons documented (6%) (Figure 5).

Hemorrhagic transformation and mortality

Hemorrhagic transformation was documented in 36 patients (20%), of whom 10 had received intravenous thrombolysis (28%). A total of 16 hospitalized patients died during their hospital stay (9%).

Predictors of hemorrhagic transformation

A logistic regression model was conducted to identify clinical factors associated with hemorrhagic transformation in patients with acute ischemic stroke. Higher stroke severity on admission, as measured by the NIHSS, was a significant predictor of hemorrhagic transformation (adjusted OR 1.06, p = 0.031), consistent with prior literature linking more severe strokes to increased hemorrhagic risk. Interestingly, intravenous thrombolysis administered in the EW was associated with a significantly lower risk of hemorrhagic transformation (adjusted OR ≈ 0.11, p = 0.014), potentially reflecting stricter patient selection criteria based on advanced neuroimaging protocols. Age, sex, conventional window thrombolysis, and mechanical thrombectomy were not significantly associated with hemorrhagic transformation in this model (Table 1).

**Table 1:** Adjusted logistic regression model showing clinical predictors of hemorrhagic transformation in patients with acute ischemic stroke (n = 182). NIHSS: National Institutes of Health Stroke Scale, CI: Confidence interval The key findings of the adjusted logistic regression model identifying clinical predictors of hemorrhagic transformation were as follows: a higher NIHSS at admission was significantly associated with higher odds of hemorrhagic transformation (AOR = 1.06, 95% CI: 1.01-1.12, p = 0.031), with each point increase corresponding to a 6% increased risk. In contrast, intravenous thrombolysis administered in the extended time window was associated with significantly lower odds (AOR = 0.11, 95% CI: 0.02-0.65, p = 0.014), indicating an 89% risk reduction. Other variables (age, sex, conventional intravenous thrombolysis, and mechanical thrombectomy) showed no statistically significant association.

Predictor	Adjusted Odds Ratio	95% CI	p-value
NIHSS on admission	1.06	[1.01 – 1.12]	0.031
IV thrombolysis (extended window)	0.11	[0.02 – 0.65]	0.014
Age	1.01	[0.99 – 1.03]	0.273
Sex (Male)	0.94	[0.44 – 2.01]	0.872
IV thrombolysis (conventional window)	0.82	[0.31 – 2.18]	0.693
Mechanical thrombectomy	1.1	[0.52 – 2.34]	0.796

Predictors of intravenous thrombolysis: multivariable and bivariate analysis

In the multivariable logistic regression model, stroke severity as measured by the NIHSS was the strongest predictor of intravenous thrombolysis administration. Compared with patients presenting with mild stroke (NIHSS 0-4), those with moderate severity (NIHSS 5-15) had significantly higher odds of receiving thrombolysis (OR = 2.4, 95% CI: 1.44-3.99, p < 0.001), and those with severe stroke (NIHSS ≥16) also showed increased odds (OR = 2.2, 95% CI: 1.19-4.10, p < 0.001). In contrast, age, sex, hypertension, and atrial fibrillation were not significantly associated with thrombolysis administration (Table 2). These findings suggest that clinical severity, rather than demographic or comorbidity profiles, played the predominant role in determining eligibility for reperfusion therapy in this cohort. 

**Table 2:** Multivariable logistic regression analysis identifying predictors of intravenous thrombolysis in patients with acute ischemic stroke. NIHSS: National Institutes of Health Stroke Scale Stroke severity, assessed using the NIHSS, was the strongest predictor of thrombolysis administration. Compared to patients with mild stroke (NIHSS 0–4), those with moderate (NIHSS 5–15) and severe strokes (NIHSS ≥16) had significantly higher odds of receiving thrombolysis. No significant associations were found for age, sex, hypertension, or atrial fibrillation. Odds ratios (OR), 95% confidence intervals (CI), and p-values are presented.

Predictor	Odds Ratio	95% CI Lower	95% CI Upper	p-value
NIHSS Moderate (5–15)	2.4	1.44	3.99	<0.001
NIHSS Severe (≥16)	2.2	1.19	4.10	<0.001
Age	1.0	0.97	1.03	0.856
Sex (Male)	1.42	0.61	3.29	0.415
Hypertension	0.67	0.28	1.61	0.369
Atrial fibrillation	1.05	0.51	2.15	0.682

A bivariate analysis was performed to evaluate the differences between patients who received intravenous thrombolysis and those who did not. Statistically significant differences were observed in infarct location (Chi-square = 9.8, p = 0.002), time from symptom onset to hospital arrival (Mann-Whitney U = 1350.5, p = 0.001), NIHSS severity (Mann-Whitney U = 1220.7, p < 0.001), performance of mechanical thrombectomy (Chi-square = 12.4, p < 0.001), and door-to-groin time (Mann-Whitney U = 1405.3, p = 0.004). In contrast, variables such as sex (Chi-square = 2.7, p = 0.263), age (Mann-Whitney U = 1605.2, p = 0.856), type 2 diabetes (Chi-square = 4.5, p = 0.339), hypertension (Chi-square = 1.7, p = 0.423), atrial fibrillation (Chi-square = 0.3, p = 0.871), prior stroke (Chi-square = 1.9, p = 0.402), smoking (Chi-square = 0.3, p = 0.845), and obesity (Chi-square = 2.1, p = 0.317) did not show significant differences in the rate of thrombolysis (Table 3).

**Table 3:** Bivariate analysis comparing patients who received intravenous thrombolysis and those who did not. Chi-square tests were applied for categorical variables (sex, diabetes, hypertension, atrial fibrillation, prior stroke, smoking, obesity, infarct location, and mechanical thrombectomy), and Mann–Whitney U tests for continuous variables (age, NIHSS on admission, onset-to-arrival time, and door-to-groin time). Statistically significant differences were observed in infarct location, time from symptom onset to hospital arrival, NIHSS severity, performance of mechanical thrombectomy, and door-to-groin time, while the remaining variables showed no significant differences. MCA: Middle Cerebral Artery, PCA: Posterior Cerebral Artery, ACA: Anterior Cerebral Artery, SCA: Superior Cerebellar Artery, PICA: Posterior Inferior Cerebellar Artery, AICA: Anterior Inferior Cerebellar Artery. M1: Sphenoidal Segment, M2: Insular Segment, M3: Opercular Segment, M4: Cortical Segment.

Variable	Test	Statistic	p-value	Significance	With intravenous thrombolysis (39)	Without intravenous thrombolysis (143)
Infarct location	Chi-square	χ² = 9.8	0.002	Significant	Localization Number of cases Left MCA 9 Left MCA M1 5 Right MCA 3 Left MCA M2 3 Right PCA 2 Left MCA M3 2 Right MCA M2 2 Isolated cases included: SCA–PICA–right PCA, left internal carotid artery, right PCA + MCA + PICA, left AICA, right MCA (M2), right MCA M3–M4, right ACA + PICA,	Localization Number of cases Left MCA 32 Right MCA 20 Left vertebral artery 8 Right PCA 6 Left PICA 5 Left cerebellar hemisphere 4 Left MCA M2 4 Left PCA4 4 Right SCA 4 Left MCA M1 (3), right vertebral artery (2), right inferior PCA (2), right PICA (2), left ACA (2), among others.
Onset-to-arrival time (minutes)	Mann–Whitney U	U = 1350.5	0.001	Significant	113.9 +/- 107.2 minutes	293 +/- 209.6 minutes
NIHSS severity	Mann–Whitney U	U = 1220.7	<0.001	Significant	Mdn: 8.0	Mdn: 4.0
Mechanical thrombectomy	Chi-square	χ² = 12.4	<0.001	Significant	11	13
Door-to-groin time (minutes)	Mann–Whitney U	U = 1405.3	0.004	Significant	176 +/- 54.8 minutes	258.5 +/- 161.3 minutes
Sex	Chi-square	χ² = 2.7	0.263	Not significant	Male: 22 patients Female: 17 patients	Male: 71 patients Female: 72 patients
Age (years)	Mann–Whitney U	U = 1605.2	0.856	Not significant	Mdn: 75 years	Mdn: 72.5 years
Type 2 diabetes	Chi-square	χ² = 4.5	0.339	Not significant	7	32
Hypertension	Chi-square	χ² = 1.7	0.423	Not significant	15	74
Atrial fibrillation	Chi-square	χ² = 0.3	0.871	Not significant	6	22
Prior stroke	Chi-square	χ² = 1.9	0.402	Not significant	2	26
Smoking	Chi-square	χ² = 0.3	0.845	Not significant	7	32
Obesity	Chi-square	χ² = 2.1	0.317	Not significant	19	65

Safety outcomes in wake-up stroke and extended window thrombolysis

In a cohort comparison, patients who received thrombolysis under extended window protocols or presented with wake-up strokes (n = 142) had similar rates of hemorrhagic transformation (19.0%) and in-hospital mortality (9.2%) compared to those treated with conventional thrombolysis (22.5% and 7.7%, respectively). These findings suggest that, in this sample, EW treatment did not confer additional short-term risk, supporting the safety of current late-window reperfusion protocols.

## Discussion

Several factors may explain the higher rate of intravenous thrombolysis observed in this study. First, the setting of a tertiary-level private hospital, which generally serves a medium-to-high socioeconomic population, may facilitate earlier recognition of ischemic stroke symptoms. This likely contributed to the fact that 47% of patients arrived within 4.5 hours of symptom onset, allowing timely reperfusion therapy.

Second, the hospital is equipped with specialized personnel, both basic and advanced neuroimaging, pharmacological therapy, and the capacity to perform mechanical thrombectomy. The importance of these resources has been emphasized in prior studies, where delayed hospital arrival was most commonly attributed to multiple prior medical evaluations before reaching a stroke center, followed by limited recognition of stroke symptoms requiring urgent treatment [22].

Another contributing factor was the use of an extended treatment window: 15% of patients were treated with intravenous thrombolysis based on EXTEND and WAKE-UP protocol criteria. Compared to earlier studies conducted before these protocols were incorporated into international guidelines, this approach expanded the pool of eligible patients. 

An important finding was that 39% of patients arriving within 4.5 hours received intravenous thrombolysis, and 33% of these also underwent adjunctive mechanical thrombectomy. In bivariate analysis, treatment rates did not differ significantly by age or baseline NIHSS score, consistent with meta-analyses demonstrating the benefits of thrombolysis regardless of these factors [23]. However, anterior circulation stroke (p = 0.012) and shorter onset-to-door time, particularly arrival within the first hour (p = 0.002), were significantly associated with treatment.

Multivariate analysis confirmed stroke severity, as measured by the NIHSS, as an independent predictor of intravenous thrombolysis administration. Compared to patients with mild stroke (NIHSS 0-4), those with moderate (NIHSS 5-15) and severe strokes (NIHSS ≥16) had more than twice the odds of receiving thrombolysis (OR 2.4 and 2.2, respectively; p < 0.001 for both). These findings align with existing literature emphasizing the prioritization of reperfusion in patients with greater neurological impairment. Other variables, including age, sex, and comorbidities such as hypertension or atrial fibrillation, were not significantly associated with treatment delivery.

The relevance of treatment times in acute stroke care is underscored by robust evidence linking earlier intravenous thrombolysis to improved functional outcomes on the modified Rankin Scale. In our study, only 18% of patients achieved a door-to-CT time <25 minutes, 21% achieved a door-to-needle time <60 minutes, and 11% achieved a door-to-groin time <120 minutes. These findings highlight a critical opportunity for process optimization.

Improvement initiatives should prioritize reducing treatment delays. Prior stepwise quality improvement studies have demonstrated that interventions such as prenotification of the stroke team before arrival, administration of alteplase in the imaging suite, and streamlined access to imaging through simplified registration can significantly reduce door-to-needle times and improve outcomes [24].

This study has several strengths, including the systematic evaluation of reperfusion practices in a high-complexity private hospital with full neurovascular capabilities, integration of extended thrombolysis protocols, and the detailed analysis of pre-hospital and in-hospital timelines. These elements provide valuable insight into stroke care performance under optimal conditions and offer a benchmark for comparison with national and international standards. 

The observed in-hospital mortality rate in this cohort was 9%, which aligns with rates reported in high-complexity settings internationally. For comparison, the Get With The Guidelines (GWTG)-stroke registry reports in-hospital mortality rates ranging from 5% to 10%, depending on stroke severity and timely access to treatment. In Mexico, the REgistro NAcional Mexicano de Enfermedad VAScular Cerebral (RENAMEVASC) study reported significantly higher in-hospital mortality in public institutions, particularly among patients with limited access to reperfusion therapies. The mortality rate observed in our study reflects the benefits of early hospital arrival, availability of advanced imaging, and rapid access to intravenous thrombolysis and mechanical thrombectomy. Nonetheless, it is important to interpret this finding cautiously, as some degree of selection or documentation bias may have influenced the reported figure.

Nonetheless, several limitations must be acknowledged. As a single-center, retrospective analysis conducted in a private tertiary facility, the findings may not be generalizable to public or lower-resource healthcare settings in Mexico. The socioeconomic profile of the patient population and the availability of specialized resources may have contributed to higher treatment rates. Additionally, key outcome measures such as functional status at discharge or follow-up (e.g., mRS) were not consistently recorded, limiting our ability to evaluate clinical effectiveness. While mortality was surprisingly low in this cohort, this should be interpreted cautiously, given the potential for selection or documentation bias.

Data integrity must also be considered as a limitation. While the dataset was carefully curated and reviewed, retrospective data collection may be subject to incomplete documentation, misclassification, or underreporting of clinical events, including complications and in-hospital mortality. In particular, the lack of systematically recorded functional outcomes and the low reported mortality raise concerns about potential information bias. These limitations highlight the need for prospective data collection frameworks and stroke registries that ensure completeness and accuracy.

Despite these limitations, the study highlights opportunities for optimizing acute stroke pathways and expanding access to timely reperfusion therapies. The findings underscore the potential impact of structural capacity, institutional protocols, and time-sensitive care models on treatment delivery. More broadly, these results support the implementation of standardized stroke pathways and quality improvement strategies across both private and public healthcare systems in middle-income countries, where disparities in stroke care remain substantial.

## Conclusions

The rate of intravenous thrombolysis observed in this study (21%) was higher than that reported in the Mexican literature. This is likely influenced by the hospital’s tertiary-level resources, the use of EW protocols, and earlier patient arrival compared with previous reports. As treatment guidelines evolve, it is expected that thrombolysis rates will continue to increase due to the inclusion of patients eligible for reperfusion in extended windows.

Patient registries for ischemic stroke are essential for systematically documenting acute-phase care, tracking quality indicators, and identifying opportunities for improvement. The use of internationally validated protocols facilitates the structured organization of hospital resources, enables certification by international associations, and promotes continuous quality improvement. Our findings highlight both the progress achieved and the challenges that remain in optimizing acute stroke care in Mexico. Ensuring broader access to intravenous thrombolysis, improving door-to-treatment times, and expanding the use of reperfusion therapies represent key areas of opportunity. Strengthening institutional and national stroke programs will be critical to improving patient-centered outcomes and reducing the burden of stroke in the Mexican population.
